# Hsp90 in the continuum of breast ductal carcinogenesis: Evaluation in precursors, preinvasive and ductal carcinoma lesions

**DOI:** 10.1186/1471-2407-10-353

**Published:** 2010-07-05

**Authors:** Flora Zagouri, Theodoros N Sergentanis, Afrodite Nonni, Christos A Papadimitriou, Nikolaos V Michalopoulos, Philip Domeyer, George Theodoropoulos, Andreas Lazaris, Effstratios Patsouris, Eleni Zogafos, Anastazia Pazaiti, George C Zografos

**Affiliations:** 1 Breast Unit, 1st Department of Propaedeutic Surgery, Hippokratio Hospital, School of Medicine, University of Athens, Greece; 2 Department of Clinical Therapeutics, Alexandra Hospital, School of Medicine, University of Athens, Greece; 3 1st Department of Pathology, School of Medicine, University of Athens, Greece

## Abstract

**Background:**

Hsp90 (heat shock protein90) is a chaperone protein essential for preserving and regulating the function of various cellular proteins. Elevated Hsp90 expression seems to be a trait of breast cancer and may be an integral part of the coping mechanisms that cancer cells exhibit *vis-à-vis *stress. This manuscript tries to examine the immunohistochemical expression of Hsp90 all along the continuum of breast ductal lesions encompassing ductal hyperplasia without atypia (DHWithoutA), atypical ductal hyperplasia (ADH), ductal carcinoma in situ (DCIS) and invasive ductal carcinoma (IDC).

**Methods:**

Tissue specimens were taken from 30 patients with DHWithoutA, 31 patients with ADH, 51 with DCIS and 51 with IDC. Immunohistochemical assessment of Hsp90 was performed both in the lesion and the adjacent normal breast ducts and lobules; the latter serving as control. Concerning Hsp90 assessment the percentage of positive cells and the intensity were separately analyzed. Subsequently, the Allred score was calculated. *Post hoc *analysis on the correlations between Hsp90 Allred score and possible predictors (grade, nodal status, tumor size, ER Allred score, PR Allred score, c-erbB-2 status and triple negative status) was conducted in IDC.

**Results:**

Hsp90 exhibited mainly cytoplasmic immunoreactivity. Hsp90 Allred score exhibited an increasing trend along the continuum of breast ductal lesions (Spearman's rho = 0.169, p = 0.031). Compared to the adjacent normal ducts and lobules, no statistically significant differences were noted in DHwithoutA, ADH and DCIS. Hsp90 expression (intensity, positive cells, Allred score) was higher in IDC, compared to the adjacent normal tissue. Higher Hsp90 expression was observed in grade 2/3 IDCs (borderline association) and tumors of larger size. At the univariable analysis, higher Hsp90 expression was associated with higher ER Allred score, PR Allred score and c-erbB-2 positivity in IDC. Triple-negative IDCs exhibited significantly lower Hsp90 expression. The multivariable logistic regression model revealed that between the three markers, solely ER Allred score and c-erbB-2 positivity were independently associated with higher Hsp90 expression in IDC.

**Conclusion:**

The above point to significant variability in Hsp90 expression with significant implications upon the effectiveness and limitations of anti-Hsp90 drugs.

## Background

Hsp90 (heat shock protein 90, also known as HSPC1 according to the most recent nomenclature assigned by the HUGO Gene Nomenclature Committee [1]) is a chaperone protein essential for preserving and regulating the function of various cellular proteins. Hsp90 mediates its actions through the formation of discrete subcomplexes, each containing proteins-cochaperones that assist protein folding and refolding during stress, protein transport and degradation [2-4]. Hsp90 interacts with a number of protein-cornerstones in breast carcinogenesis. Hsp90 counterparts include estrogen receptors (ER), p53 protein, hypoxia-induced transcription factor HIF-1alpha, protein kinase Akt, Raf-1 MAP kinase and a variety of receptor tyrosine kinases, such as erbB2 (reviewed in [5]).

Elevated Hsp90 expression seems to be a trait of breast cancer and may be an integral part of the coping mechanisms that cancer cells exhibit *vis-à-vis *stress [6-8]. As a result clinical implications of Hsp90 expression have emerged; Hsp90 upregulation may be a sign of unfavourable prognosis [9]. Another clinical implication concerns the pharmacological inhibition of Hsps [10-14]. Indeed, positive results have appeared in the literature concerning 17-allylamino, 17-demethoxygeldanamycin (17-AAG), the first Hsp90 inhibitor having entered clinical trials [5,15].

Previous studies have appeared concerning IDC [9,16], where positive association between Hsp90 expression and grade, nodal positivity, tumour size, ER, c-erbB-2 and decreased survival have been documented [9]. However, Hsp90 expression in precursor (ADH) and preinvasive lesions (DCIS) have not yet been studied. Precursor and preinvasive lesions exhibit a distinct immunohistochemical profile regarding established markers such as ER, progesterone receptors (PR), c-erbB-2 etc [17]. It seems thus fairly rational to postulate that precursor and preinvasive lesions may also exhibit a distinct profile of Hsp90 expression. Interestingly, the sole study to focus exclusively on precursor lesions concerns the lobular series [18], where Hsp90 downregulation became unexpectedly evident.

Given the above, this is the first study to examine the immunohistochemical expression of Hsp90 all along the continuum of breast ductal lesions encompassing ductal hyperplasia without atypia (DHWithoutA), ADH, DCIS and IDC. In addition, a *post hoc *sub-analysis in IDC incorporating ER, PR, c-erbB-2 (integrated as triple negative status and assessed through a multivariable approach, for the first time to our knowledge), tumor size, grade and nodal status is presented.

## Methods

This study included formalin-fixed, paraffin-embedded tissue specimens from 30 patients with DHWithoutA, 31 patients with ADH, 51 with DCIS and 51 patients with IDC. The patients' age at operation ranged between 31 and 78 (median age: 51 years). The diagnosis of the lesions was established by vacuum-assisted breast biopsy, excisional breast biopsy, lumpectomy and modified radical mastectomy. All lesions were independent i.e., no DHWithoutA, ADH, DCIS lesions coexisted with invasive carcinomas. Cases coexisting with lobular neoplasia and infiltrating lobular carcinoma were also excluded.

Hsp90 was immunohistochemically detected using the mouse monoclonal antibody Hsp90 (clone JPB24, NCL-Hsp90, Novocastra supplied by Menarini). The dilution was 1:500 and the incubation time was equal to 18 h (at 4°C). The visualization was performed using an avidin-biotin detection system m (Envision, Dako). Antigen retrieval was achieved in 0.01 M citrate buffer (pH = 6.0) at 85°C for 15 min. Immunohistochemical assessment of Hsp90 was performed both in the lesion and the adjacent normal breast ducts and lobules; the latter serving as control. Negative controls were assessed by omitting the primary antibody.

Concerning Hsp90 assessment: i) the percentage of positive cells and ii) the intensity was separately analyzed. Subsequently, the Allred score was appropriately calculated summing the proportion score (represented by the estimated proportion of positive cells), as well as the intensity score (represented by the average intensity of positive cells) [19].

For immunohistochemistry (IHC), the following antibodies were used: PR (636, Dako), ER (ID5, Dako) and c-erbB-2 (CB11, Novocastra™). Sections (4 μm thick) cut from formalin-fixed paraffin embedded tissue were used. After deparaffinization in xylene and hydration in graded ethanol solutions, the sections of IDC tissue were subjected to pretreatment in order to enhance antigen retrieval. The EnVision + System-HRP (DAB) (DakoCytomation, Glostrup, Danemark) was used with primary antibodies against the following antigens: PR, ER and c-erbB-2. Immunohistochemistry was performed according to the protocols provided by the manufacturer. Concerning the immunohistochemical expression of ER and PR, both the intensity (negative, 1+ to 3+) and percentage of immunopositive cells were evaluated. Subsequently, the Allred score was calculated [19].

The expression of c-erbB-2 was assessed as follows: i) negative, when no staining was documented or when membrane staining was present in less than 10% of tumor cells, ii) weak staining (+), when partial membrane staining was documented in more than 10% of tumor cells, iii) moderate staining (++) when weak/moderate complete membrane staining was present in more than 10% of tumor cells and iv) strong staining when strong, complete membrane staining was observed in more than 10% of tumor cells. Cases with negative and weak c-erbB-2 staining were considered as negative, whereas cases with strong c-erbB-2 staining were considered as positive. In cases with moderate staining, CISH (chromogenic in situ hybridization) was performed; subsequently these cases were considered as negative or positive. The ready-to-use Spot Light HER2/neu DNA Probe (Zymed/InVitrogen, San Fransisco, USA) was used for CISH; this digoxygenin-labeled probe is located on 17q12-21 and covers the entire gene area.

In all cases, ten fields (×40 magnification) were assessed and a minimum of 100 cells were evaluated in the designated areas, so as to evaluate the lesion as a whole. The immunohistochemical evaluation was performed independently by two consultant histopathologists (AN and AL).

The intensity was treated as an ordinal variable-score (0: negative, low: 1, moderate: 2, high: 3); Allred scores (ER, PR, Hsp90) have been treated as ordinal variables, whereas tumor size has been treated as a continuous variable. Due to deviation from the normal distribution, non-parametric statistics were chosen. The analysis included two steps: i) comparison of Hsp90 expression (Hsp90 intensity, positive cells (%), Allred score) in the lesions *vs*. the adjacent normal ducts and lobules. Where appropriate, power calculations were performed. ii) A test for significance of the Hsp90 trend along the continuum of lesions. The correlation between Hsp90 Allred score in the lesion and the severity of the lesion (1:DHWithoutA, 2:ADH, 3:DCIS, 4:IDC) was evaluated; Spearman's rank correlation coefficient was appropriately implemented. iii) *Post hoc *analysis on the correlations between Hsp90 Allred score and possible predictors (grade, nodal status, tumor size, ER Allred score, PR Allred score, c-erbB-2 status and triple negative status) was conducted in IDC. Once again non-parametric statistics were performed under the light of deviation from normality and limited sample size. In addition, concerning the three immunohistochemical markers, multivariable logistic regression was performed as follows: Hsp90 Allred score was converted to a binary dependent variable [0: Hsp90 Allred score ≤ 7 (n = 28) and 1: Hsp90 Allred score = 8 (n = 23), as the median Hsp90 Allred score was equal to 7], whereas ER Allred score (continuous variable), PR Allred score (continuous variable) and c-erbB-2 status (positive *vs*. negative) were the independent variables.

Results of borderline statistical significance (0.1 < p < 0.05) have also been reported, given that they might become significant in the context of a larger sample size. Statistical analysis was performed with STATA 8.0 statistical software (Stata Corporation, College Station, TX, USA).

Informed consent was obtained by all participants in this study. This study has been approved by the local Ethics Committee, in compliance to the Helsinki Declaration.

## Results

**Figure 1:**
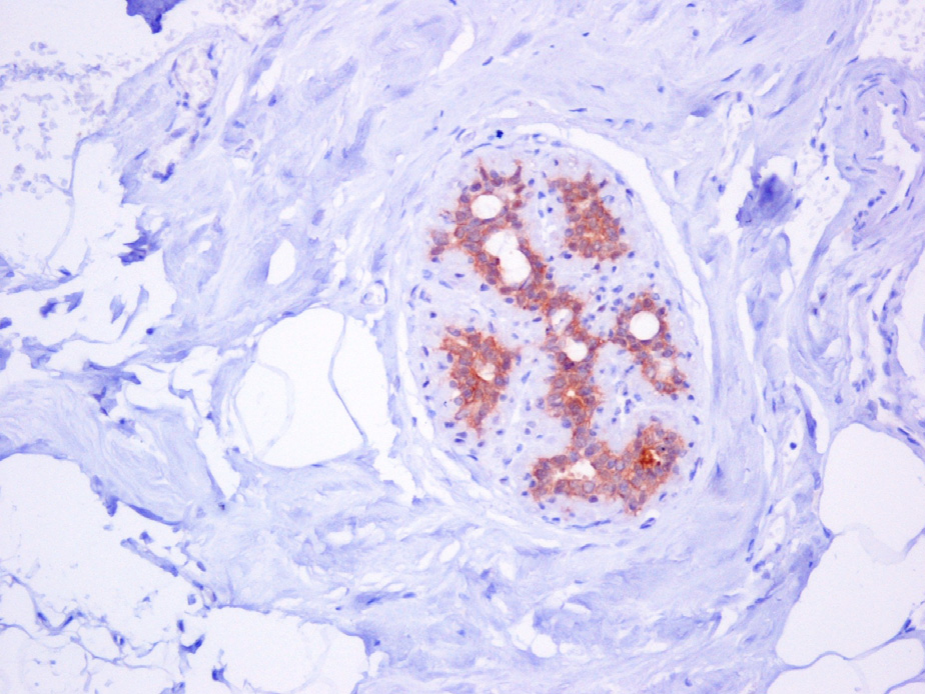
**Moderate Hsp90 immunostaining in epithelial cells of normal breast (ducts and lobules) (×200)**.

**Figure 2:**
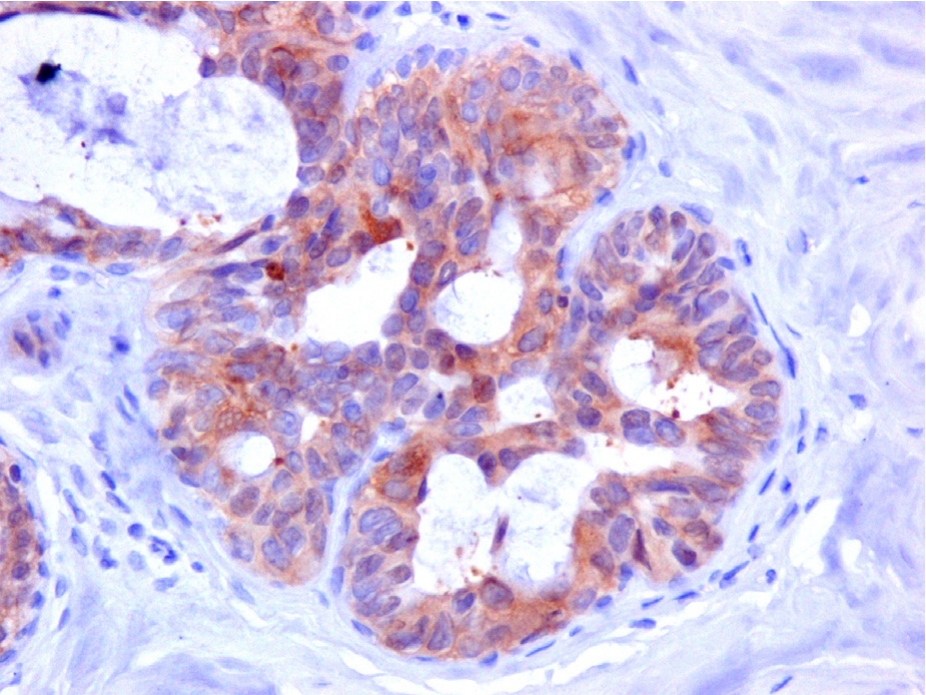
**Moderate expression of Hsp90 in atypical ductal hyperplasia (×400)**.

**Figure 3:**
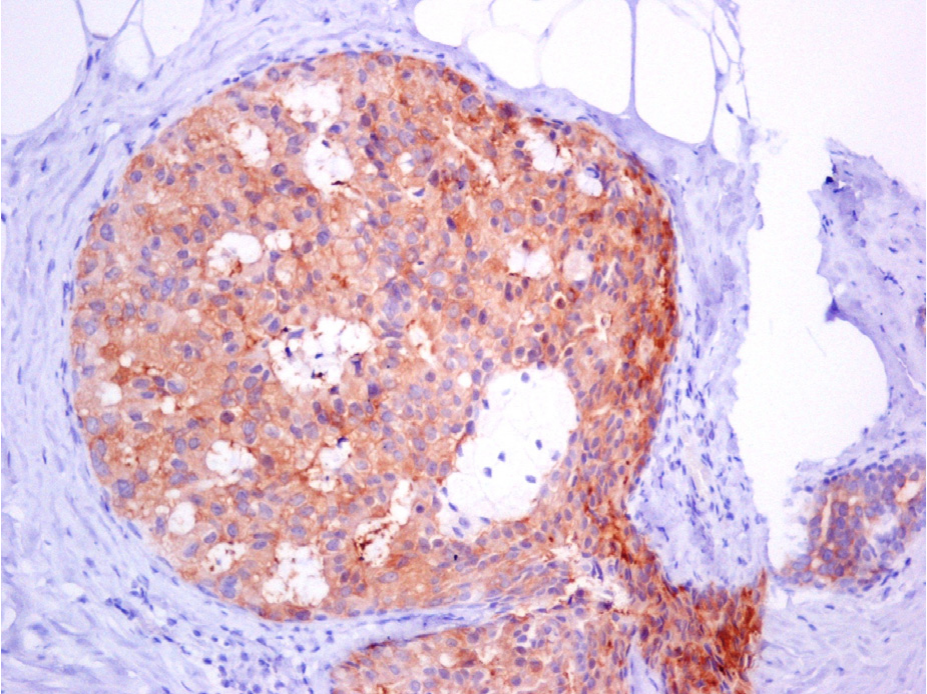
**In situ ductal carcinoma with moderate expression of Hsp90 (×200)**.

**Figure 4:**
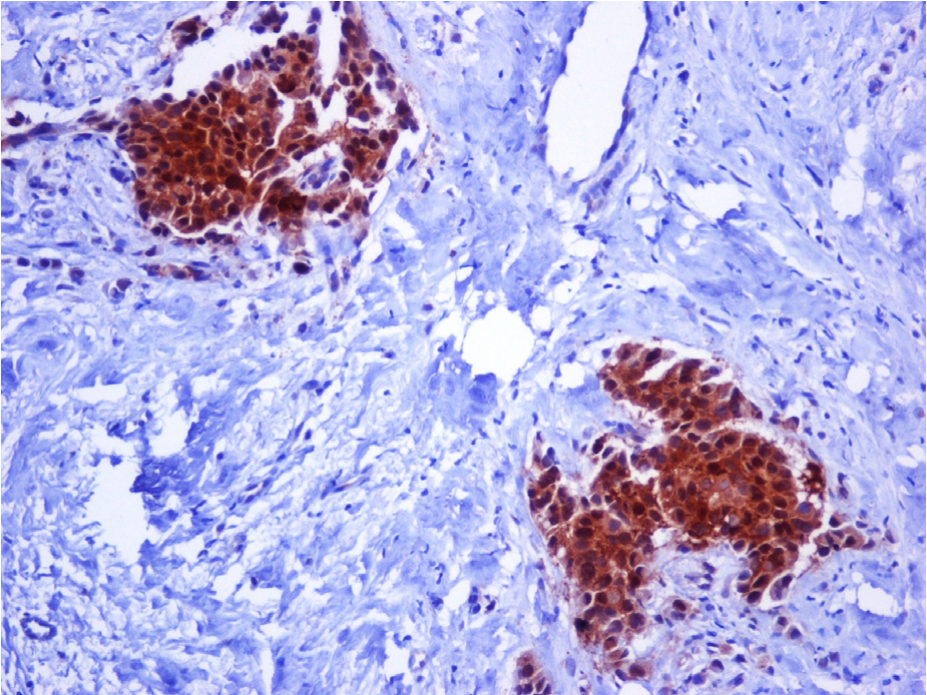
**Intense expression of Hsp90 in invasive ductal carcinoma of the breast (×200)**.

**Figure 5:**
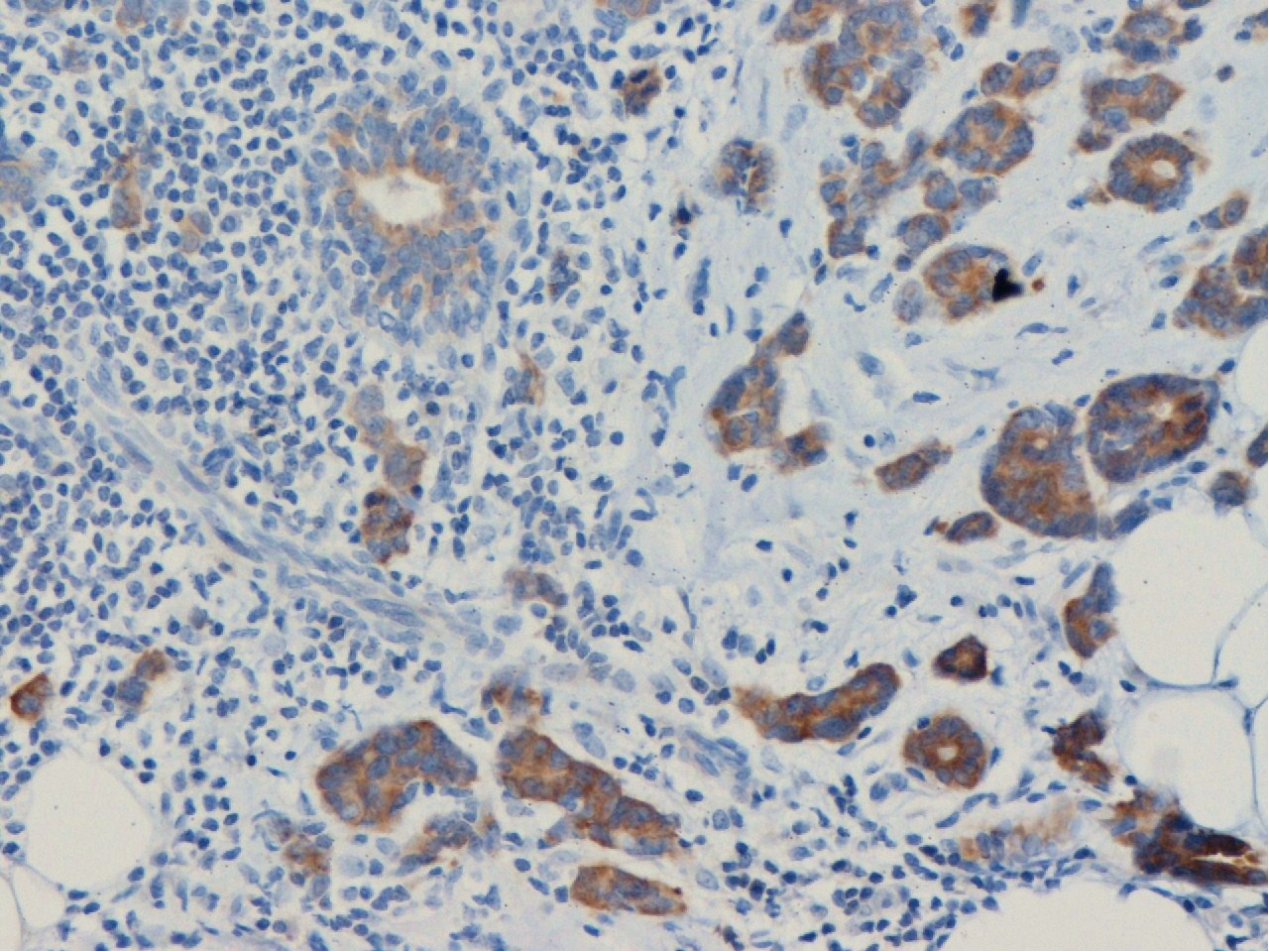
**Both the percentage and the staining intensity are increased in invasive ductal carcinoma in comparison with the normal ductile (×200)**.

Hsp90 exhibited mainly cytoplasmic immunoreactivity in epithelial cells of normal breast (ducts and lobules) (figure 1), ADH (figure 2), DCIS (figure 3) and IDC (figure 4, 5). Some epithelial cells also showed nuclear staining; nevertheless, all the DHWithoutA, ADH, DCIS and IDC foci mainly presented with a positive cytoplasmic immunoreaction for Hsp90. The percentage of these positive cells and the staining intensity were evaluated.

The percentage of Hsp90 positive cells, the intensity of Hsp90 staining, as well as the Allred score are presented in detail in Table 1. Compared to the adjacent normal ducts and lobules, no statistically significant differences were noted in DHWithoutA and ADH. Despite the numerical superiority in DCIS, the discrepancies failed to reach statistical significance. Indeed, given the present results, and assuming a type I error equal to 0.1 (threshold of borderline statistical significance), the study power was equal to 0.21 i.e. 21%. Given the present results and under the same assumptions, 466 cases of DCIS would be needed to achieve the optimal study power of 80%. Hsp90 expression (intensity, positive cells, Allred score) was higher in IDC, compared to the normal adjacent ducts and lobules (Table 1). Hsp90 Allred score exhibited an increasing trend along the continuum of breast ductal lesions (Spearman's rho = 0.169, p = 0.031).

**Table 1:** Hsp90 expression in ADH, DCIS and IDC

	Adjacent normal ducts and lobules	DHwithoutA (n = 30)	p^a^
**Hsp90 intensity (score)**	2.27 ± 0.58	2.33 ± 0.55	0.593
**Hsp90 positive cells (%)**	70.7 ± 9.44	71.7 ± 12.3	0.664
**Hsp90 Allred score**	7.00 ± 0.87	7.00 ± 0.79	0.896
			

	**Adjacent normal ducts and lobules**	**ADH (n = 31)**	**p^a^**

**Hsp90 intensity (score)**	2.26 ± 0.63	2.39 ± 0.62	0.346
**Hsp90 positive cells (%)**	69.4 ± 10.3	72.3 ± 14.7	0.195
**Hsp90 Allred score**	6.97 ± 0.98	7.00 ± 0.97	0.660
			

	**Adjacent normal ducts and lobules**	**DCIS (n = 51)**	**p^a^**

**Hsp90 intensity (score)**	2.29 ± 0.61	2.43 ± 0.67	0.285
**Hsp90 positive cells (%)**	69.0 ± 8.5	71.0 ± 14.9	0.298
**Hsp90 Allred score**	7.00 ± 0.77	7.14 ± 0.94	0.360
			

	**Adjacent normal ducts and lobules**	**IDC (n = 51)**	**p^a^**

**Hsp90 intensity (score)**	2.20 ± 0.63	2.49 ± 0.54	**0.018**
**Hsp90 positive cells (%)**	68.8 ± 9.7	74.3 ± 14.9	**0.004**
**Hsp90 Allred score**	6.90 ± 0.78	7.33 ± 0.77	**0.002**

^a^p-value derived from Wilcoxon matched-pairs signed-ranks test

In the subanalysis concerning clinicopathological features (Table 2), higher Hsp90 expression was observed in grade 2/3 IDCs (borderline association), and larger tumor size. Table 2 presents the results in detail; median tumor size was equal to 2 cm and the dichotomization to cases < median and ≥median are provided for purely descriptive purposes, given that tumor size was treated as a continuous variable.

**Table 2:** Subanalysis in IDC cases: clinicopathological variables

Variables	N (%)	Hsp90 Allred score in IDC	p
Grade			**0.061^MWW^**
*Grade 1*	3 (5.9%)	6.67 ± 0.58	
*Grade 2 & 3*	48 (94.1%)	7.38 ± 0.76	
Nodal status			0.392^S^
*N0*	29 (56.9%)	7.28 ± 0.84	
*N1*	13 (25.5%)	7.23 ± 0.73	
*N2*	3 (5.9%)	7.67 ± 0.58	
*N3*	6 (11.7%)	7.67 ± 0.52	
Tumor size^§^			**0.033^S^**
*< median*	20 (39.2%)	7.10 ± 0.97	
*≥median*	31 (60.8%)	7.48 ± 0.57	

^MWW^p-value derived from Mann-Whitney-Wilcoxon test for independent samples; ^S^p-value derived from Spearman's rank correlation coefficient; ^§ ^tumor size has been treated as a continuous variable and thus the two subcategories serve purely descriptive purposes

**Figure 6:**
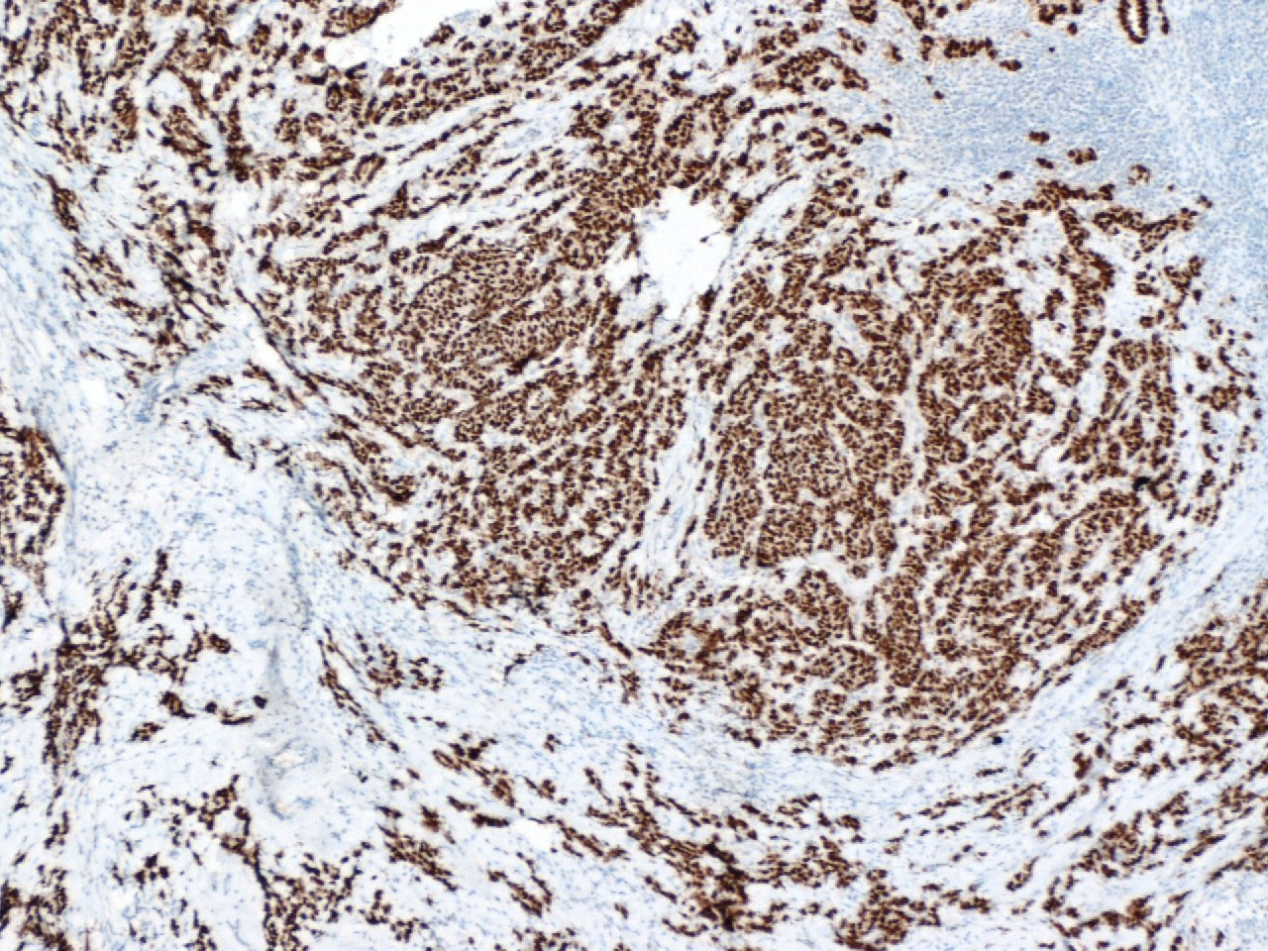
**ER strong intensity in invasive ductal carcinoma of the breast (×50)**.

**Figure 7:**
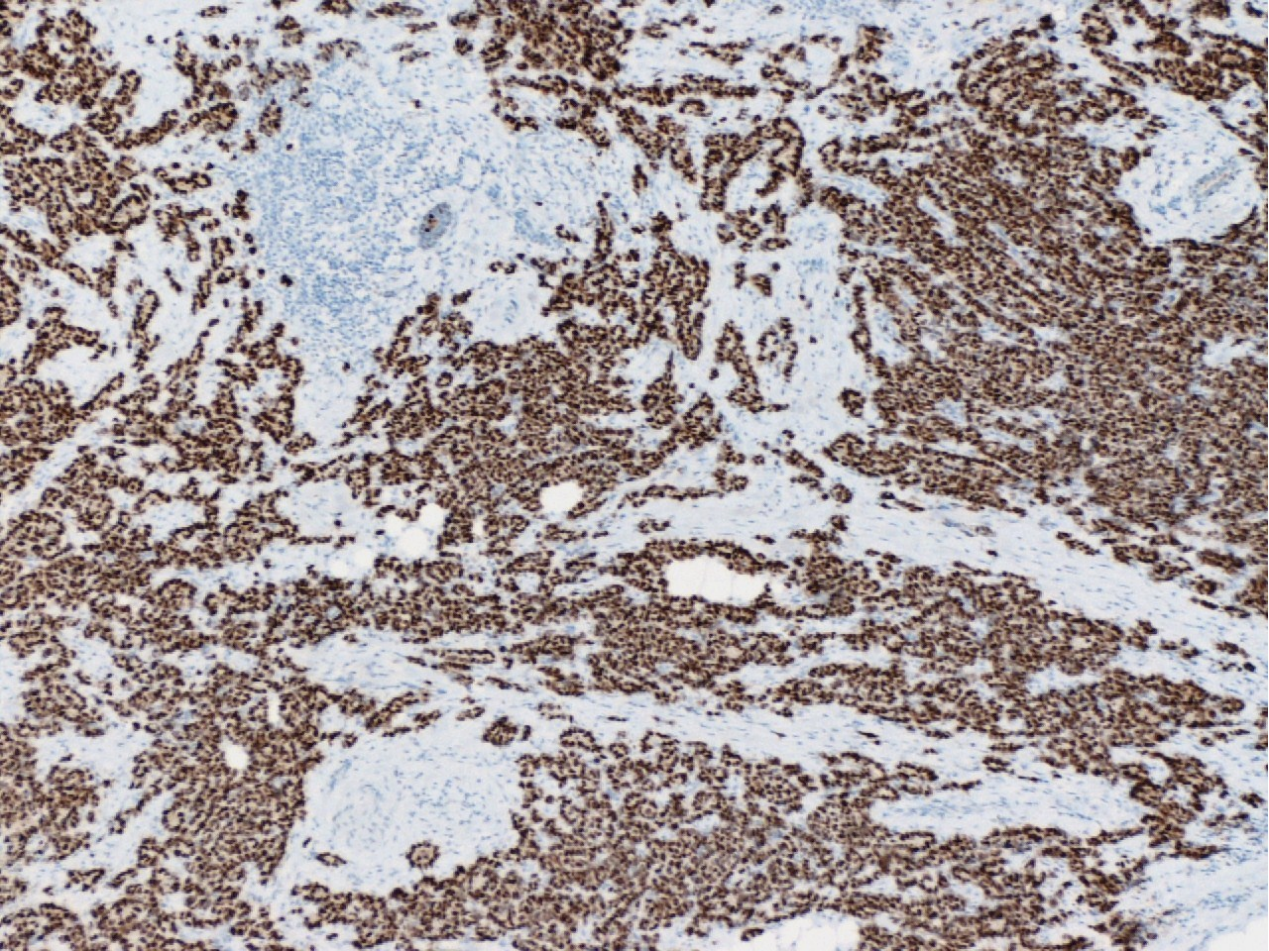
**PR strong intensity in invasive ductal carcinoma of the breast (×50)**.

**Figure 8:**
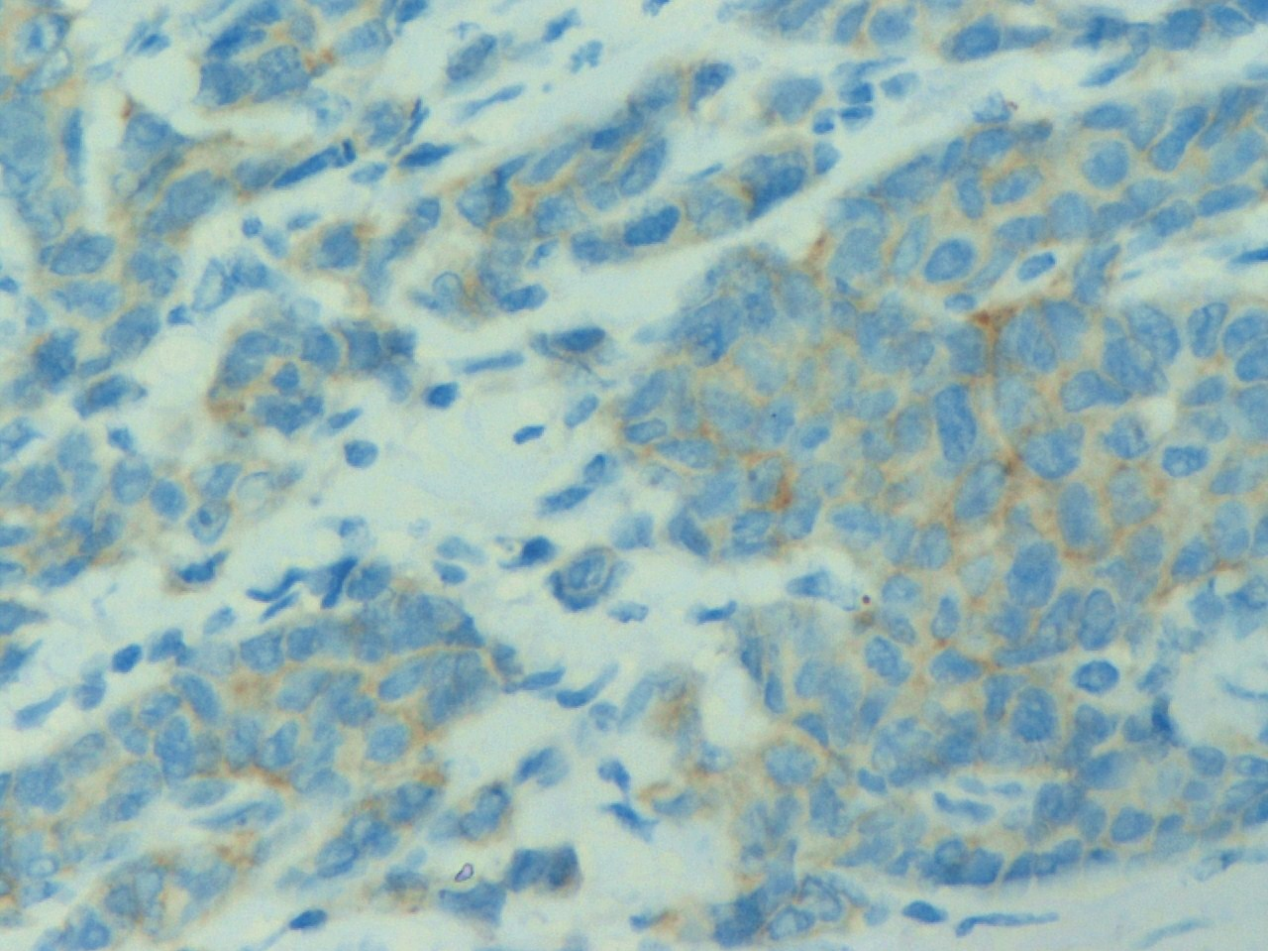
**c-erbB2 no membranous staining in invasive ductal carcinoma of the breast (×400)**.

Table 3 presents the associations of Hsp90 Allred score with the immunohistochemical markers (figure 6, 7, 8). At the univariable analysis, higher Hsp90 expression was associated with higher ER Allred score (median: 7), PR Allred score (median:4) and c-erbB-2 positivity. Interestingly, triple-negative IDCs exhibited significantly lower Hsp90 expression (of note, the nodal status of triple-negative IDCs did not differ from the remaining tumors, p = 0.391, Mann-Whitney-Wilcoxon test for independent samples [MWW]) present It is worth mentioning that ER Allred score was positively associated with PR Allred score (Spearman's rho = 0.522, p = 0.0001), whereas no significant association existed between ER Allred score and c-erbB-2 status (p = 0.849, MWW) nor between PR Allred score and c-erbB-2 status (p = 0.610, MWW).

**Table 3:** Subanalysis in IDC cases: immunohistochemical variables

Univariable analysis			
**Variables**	**N (%)**	**Hsp90 Allred score**	**p**

Triple-negative status^§^			**0.0004^MWW^**
*Triple-negative*	9 (17.7%)	6.44 ± 1.01	
*Non-triple-negative*	42 (82.3%)	7.52 ± 0.55	
ER Allred score^†^			**0.004^S^**
*< median*	23 (45.1%)	7.04 ± 0.93	
*≥median*	28 (54.9%)	7.57 ± 0.50	
PR Allred score^†^			**0.020^S^**
*< median*	21 (41.2%)	6.95 ± 0.86	
*≥median*	30 (58.8%)	7.60 ± 0.56	
c-erbB-2 status			**0.011^MWW^**
*Negative*	29 (56.9%)	7.10 ± 0.86	
*Positive*	22 (43.1%)	7.64 ± 0.49	

**Multivariable analysis**			

**Variables**	**Category or increment**	**OR (95% CI)**	**p**

ER Allred score	1 point increase^‡^	1.29 (0.99-1.66)	**0.055**
PR Allred score	1 point increase	1.18 (0.92-1.51)	0.195
c-erbB-2 status	Positive vs. negative	1.85 (1.14-3.02)	**0.013**

^MWW^p-value derived from Mann-Whitney-Wilcoxon test for independent samples; ^S^p-value derived from Spearman's rank correlation coefficient; ^§ ^not included in the multivariable analysis, as it integrates all three components (ER, PR, c-erbB-2); ^†^ER Allred score, PR Allred score have been treated as continuous variables and thus the subcategories serve purely descriptive purposes ^‡^practical interpretation: increase in Allred score by one unit predicts OR = 1.29.

The multivariable logistic regression model revealed that between the three markers, solely ER Allred score (OR = 1.29, 95%CI: 0.99-1.66) and c-erbB-2 positivity were independently associated with higher Hsp90 expression (OR = 1.85, 95%CI: 1.14-3.02).

## Discussion

This study is the first to investigate Hsp90 expression in precursor and preinvasive lesions. Noticeably, ADH did not exhibit a statistically significant difference whereas the numerical difference pointing to Hsp90 upregulation in DCIS failed to reach statistical significance. This finding may imply that the precursor context may not entail the cellular stress present in invasive cancer [18]. Consequently, as an integral part of the stress response, Hsp90 *per se *appears not to have been triggered early. Nevertheless, the steady numerical increase in Hsp90 Allred score along the ductal continuum may point to a simultaneous underlying increase in cellular stress, as well as to the multifaceted role mediated by Hsp90 in regulating the stability and function of many oncogenic and anti-oncogenic proteins, such as (ER), p53 protein, hypoxia-induced transcription factor HIF-1alpha, protein kinase Akt, Raf-1 MAP kinase and a variety of receptor tyrosine kinases, such as erbB2 [3-5].

At the end of the continuum, the gradual increase in Hsp90 expression reached statistical significance; Allred score and both its components (intensity and percentage of positive cells) were increased in IDC. Within the IDC group a variety of inherent tumour features seemed to correlate with higher Hsp90 expression. Tumors of larger size also presented with more pronounced Hsp90 upregulation, which is in accordance with previous reports [9] and seems to reflect the stress-related events in rapidly proliferating hypoxic (due to their size) tumours. Concerning tumor grade, however, the positive association with Hsp90 immunostaining should be interpreted with caution, given its borderline nature, as well as the small number of grade 1 IDCs in our sample.

Concerning immunohistochemical markers, this is the first study to adopt a multivariable approach. The univariable analysis shows that the higher Hsp90 expression was associated with higher ER, PR expression and c-erbB-2 positivity. The association between PR and Hsp90 expression is in line with previous studies [20,21]; nevertheless, given that ER and PR were closely associated in our study sample, as expected [22], this represented a secondary indirect association. As a result, the multivariable approach reported that the sole immunohistochemical markers independently associated with elevated Hsp90 expression are ER and c-erbB-2. This confirms and adds to the validity of the findings reported by Pick et al. [9], who however only performed a univariable analysis.

Commenting further on the immunohistochemical profile, significantly decreased Hsp90 expression has been observed in triple-negative tumours. This is in accordance with the study by Sun et al [16] and may seem fairly rational as Hsp90 is associated with ER and c-erbB-2 expression. Our study highlights a paradox; on the one hand elevated Hsp90 has been suggested as a poor prognostic factor [9], whilst on the other hand triple-negative tumors, exhibiting *decreased *Hsp90 expression, are associated with poor prognosis (reviewed in [23]). Under the light of the above, the application of Hsp90 as a poor prognostic factor in all types of breast cancer may be worth interpreting with caution; accordingly, no statistically significant association emerged between nodal status and Hsp90 expression in our study, contrary to the report by Pick et al [9]. Nevertheless, the present study is not a prognosis study and thus no further firm conclusions can be drawn on this important field. Future studies focusing exclusively on triple negative tumors seem desirable, as triple-negative tumors were solely 9 (17.7%) of all IDCs in this study.

The evaluation of Hsp90 expression all along the ductal continuum, presented herein, may have particular clinical significance. Firstly, given that ADH did not exhibit marked Hsp90 upregulation, the potential involvement of anti-Hsp90 drugs as chemoprevention agents may not be supported. In addition, given the associations between Hsp90, ER and c-erbB-2, it seems desirable that the effects of Hsp90 targeting drugs should be evaluated separately on different immunohistochemical types of IDC, since their effectiveness may vary accordingly.

In our cases, some epithelial cells of DHWithoutA, ADH, DCIS and IDC showed scarce nuclear Hsp90 localization. It should be declared that nuclear staining was not taken into account at the calculation of Allred score, as the latter was based exclusively on cytoplasmic Hsp90 staining. At any case, the significance of nuclear Hsp90 expression remains elusive, as some studies have not documented any nuclear Hsp90 expression in invasive ductal carcinomas [9], whereas other researchers have [24]. Other researchers have correlated nuclear Hsp90 staining with MHC class I expression in invasive breast carcinomas [25].

Concerning the limitations of this study, a number of statistical points should be noted. Firstly, although this study comprises the whole spectrum of ductal carcinogenesis, the lack of power may have inhibited the documentation of significant associations; for instance numerical discrepancies concerning DCIS may become significant in larger comparative studies (approximately 500 DCIS cases). Moreover, the limited sample size did not permit the performance of a multivariable analysis including both clinicopathological and immunohistochemical features, as the number of observations per cell was not sufficient. Nevertheless, this study is the first to adopt a multivariable assessment of Hsp90 predictors. On the other hand, despite the relatively small sample size, significant associations, such as the ER/Hsp90 and c-erbB-2/Hsp90 interplay, have emerged and persisted at the multivariable approach; this may point to their reproducibility in larger samples. Nevertheless, further studies with larger number of patients are warranted.

Other limitations of this study pertain to the method performed (i.e., immunohistochemistry); the lack of an automated quantitative procedure may have clouded some associations. To ensure the objectivity of the assessment, the percentage and intensity were assigned by two independent pathologists blind to one another's results; however, the need of additional verification of the present results through Western blot should be declared. Moreover, all comparisons have been made versus the adjacent normal ducts and lobules, as previously described [18]; it would be of interest to verify the present results also on normal glands taken for example from reduction mammoplasties or adjacent to fibroadenomas. In addition, future studies should examine whether the findings of the subanalysis on IDC (i.e., those implicating ER, PR and c-erb-B2) are reproducible in DHWithoutA, ADH or DCIS; data on these subpopulations were not available in this study. Finally, reporting the survival would yield additional information concerning the clinical significance of Hsp90 expression; the design of this retrospective study did not include follow-up for survival analysis.

## Conclusion

In conclusion, DHWithoutA, ADH and DCIS do not exhibit marked Hsp90 upregulation, while IDC present with elevated Hsp90 expression. Within IDC, a higher grade, larger tumor size, higher ER expression and c-erbB-2 positivity correlated with higher Hsp90 expression. Noticeably, triple-negative tumours exhibited decreased Hsp90 expression. The above point to significant variability in Hsp90 expression with significant implications upon the effectiveness and limitations of anti-Hsp90 drugs.

## Competing interests

The authors declare that they have no competing interests.

## Authors' contributions

All authors read and approved the final manuscript.

FZ: conceived the idea, participated in the design of the study and assisted in the writing of the manuscript; TNS: participated in the design of the study, performed the statistical analysis and assisted in the writing of the manuscript; AN: performed the pathological evaluation; CAP: evaluated critically the manuscript; NVM: performed vacuum-assisted breast biopsy and open surgery; PD: performed vacuum-assisted breast biopsy and open surgery; GT: performed vacuum-assisted breast biopsy and open surgery; AL: performed the pathological evaluation; EP: evaluated critically the manuscript; EZ: revised and evaluated critically the manuscript; AP: performed vacuum-assisted breast biopsy and open surgery, evaluated critically the manuscript; GCZ: conceived of the study, participated in its design, performed vacuum-assisted breast biopsy and open surgery and evaluated critically the manuscript

## Pre-publication history

The pre-publication history for this paper can be accessed here:

http://www.biomedcentral.com/1471-2407/10/353/prepub
